# Preparation of Multi-Walled Carbon Nanotube/Amino-Terminated Ionic Liquid Arrays and Their Electrocatalysis towards Oxygen Reduction

**DOI:** 10.3390/ma3010672

**Published:** 2010-01-25

**Authors:** Zhijuan Wang, Rose-Marie Latonen, Carita Kvarnström, Ari Ivaska, Li Niu

**Affiliations:** 1 Laboratory of Analytical Chemistry, Process Chemistry Centre, Åbo Akademi University, Biskopsgatan 8, FI-20500 Turku/Åbo, Finland; 2 State Key Laboratory of Electroanalytical Chemistry, Changchun Institute of Applied Chemistry, and Graduate University of the Chinese Academy of Sciences, Chinese Academy of Sciences, Changchun 130022, China

**Keywords:** multi-walled carbon nanotube, arrays, ionic liquid, electrocatalysis

## Abstract

Arrays of aligned multi-walled carbon nanotube-ionic liquid (MIL) were assembled on silicon wafers (Si-MIL). Formation of Si-MIL was confirmed by FTIR, AFM and Raman techniques. The electrochemical measurements indicated that Si-MIL showed good electrocatalysis towards oxygen reduction compared with MIL drop-cast on a glassy carbon electrode.

## 1. Introduction

The unique properties of carbon nanotubes (CNTs) have been the reason for the rapid progress in synthesis of nanotubes and the use of CNTs in electronics [1]. Numerous studies have indicated that carbon nanotubes have several good electrochemical properties. For example, multi-walled carbon nanotubes (MWCNTs) are electrocatalytically active in the reduction of oxygen [2]. Although the potential of oxygen reduction is lower than with other carbon electrodes, the reduction potential is still rather high -0.4 V [3]. Therefore, the electrocatalytic properties of MWCNTs should further be improved, e.g. by functionalization with some other materials. 

Our previous results have indicated that modification of single-walled carbon nanotubes (SWCNT) by amino-terminated ionic liquids (1-(3-aminopropyl)-3-methylimidazolium bromide, IL-NH_2_) do not affect their electrocatalytical properties towards oxygen reduction [4], but the presence of IL-NH_2_ was found to favor the electrocatalytic oxidation of NADH [5]. Moreover, modification of CNTs with IL-NH_2_ improved their compatibility and stability, creating more opportunity for applications of CNTs in sensors and actuators by improving the electrical contact with bulky media. More interestingly, IL-NH_2_-modified CNTs exhibited switchable solubility, high charge-transfer activity and high electronic conductivity. Reduction of oxygen was also facilitated at the IL-NH_2_-modified CNT electrode [6]. Therefore, also in this work, we chose to modify CNTs by covalent bonding with IL-NH_2_.

On the other hand, it has been reported that the edges-plane sites and tube ends of CNTs are the reactive sites [7,8]. Several reports indicate that the end group functionalities are indeed responsible for the electrochemical activity of CNTs [9,10]. In order to fully utilize the outstanding electronic properties of CNTs, the focus of research so far has been on the growth of vertically well-aligned carbon nanotube arrays on suitable substrates [11]. The highly oriented CNT structure is crucial to improve the performance of CNTs. High degree of orientation of the electrode material, in general, always increases the activity of the material, e.g., oriented polyaniline nanotube array was found to be excellent electrode material for ultrasensitive detection of nucleic acid [12]. The densely arrayed but mutually separated CNTs on a conductive substrate are ideal in constructing high-surface-area electrodes for fuel cell application and in developing amperometric chemical sensors and biosensors [13]. The aligned carbon nanotube structures have been obtained by using chemical vapor deposition (CVD) [11,13,14,15,16,17], electron-beam lithography (EBL) [18] and electrospinning [19]. However, CVD process requires high temperature (>800 °C) and is therefore not applicable to devices with limited stability [14,15,16,17,19]. The EBL has its inherent limitations such as limited pattern area, low throughput and high production costs [18]. Electrospinning is an electrostatic method for the fabrication of long organic fibers [8]. Carbon nanotube-polymer composite fibers have also been fabricated by using this technique. Although the nanotubes are considered to have fibril structure, they are not aligned macroscopically because the fibers are randomly distributed [19]. Therefore, the wet chemical method [20,21] was used to assemble CNTs. This method is simple and the reaction conditions are gentle. On the other hand, the surface coverage can also be changed by controlling the density of the –OH groups on the surface of the silicon substrate, which can be done by controlling the oxidation treatment of the silicon substrate. The carbon nanotubes have also successfully been assembled on the surface of gold substrate through covalent bonding [19]. 

Electrochemical reduction of oxygen is also of great fundamental and practical importance in development of fuel cells [22] and in the detection of O_2_ levels in such research areas as biochemistry, neuroscience, and physiology and studying fish cultures [23]. Therefore it is very important to develop materials and methods for easy reduction of oxygen. In this study we have focused on development of an array of aligned multi-walled carbon nanotube-ionic liquid (MIL) assembled on silicon wafer (Si-MIL) as the electrode for catalytic reduction of oxygen. 

Based on the discussion above we have, in this paper, studied the process of assembling MWCNTs into arrays at the surface of silicon wafer by a wet chemical method. IL-NH_2_ was then attached to the out-sticking end of the MWCNTs, and the resultant layer was tested as catalytic agent for reduction of oxygen. After functionalization with IL-NH_2_ the ends of the multi-walled carbon nanotubes will be positively charged and the electrostatic repulsion between the individual modified CNTs prevents aggregation of the carbon nanotubes.

## 2. Experimental

### 2.1. Materials 

1-Methylimidazole (99.0%, Fluka) was distilled under reduced pressure before use. 3-Bromopropylamine hydrobromide (98%, Aldrich), ethanol (~96%, Fluka), dicyclohexylcarbodiimide (DCC, 99%, Aldrich), dimethylformamide (DMF, 99.8%, Fluka), HNO_3_ (65%, J.T. Baker), H_2_SO_4_ (95–97%, J.T. Baker), H_2_O_2_ (30%, Merck kGaA 64271 Darmstadt, Germany), HCl (37%, Sigma-Aldrich), NH_3_ (25%, Merck kGaA 64271 Darmstadt, Germany), and ethyl acetate (99.5%, Fluka) were used as received. MWCNTs prepared by CVD were purchased from Shenzhen Nanotech Port Ltd. Co. (China). All aqueous solutions were prepared with ultrapure water (>18 MΩ) obtained from a Milli-Q Plus system (Millipore).

### 2.2. Purification of MWCNTs

MWCNTs require surface activation before nanoclusters can be attached to a surface. The pristine MWCNTs were purified and activated according to the method developed by Li and Grennberg [24]. The as-received MWCNTs were treated with a 1:3 v/v mixture of HNO_3_ and H_2_SO_4_ at 50 °C for 5 h with continuous ultrasonication (20W). The product was centrifuged, washed with ethanol, and dried overnight under vacuum at 60 °C.

### 2.3. Synthesis of IL-NH_2_

IL-NH_2_ was prepared according to the procedure described in reference [25]. Briefly, 3-bromopropylamine hydrobromide (1.10 g, 5 mmol) and 1-methylimidazole (0.395 mL, 5 mmol) were added to 12.5 mL ethanol, forming a colorless solution which was refluxed under nitrogen for 24 h. The resulting turbid mixture was purified by re-crystallization from ethanol, with ethyl acetate as anti-solvent. Finally, the resulting white powder was dried overnight under vacuum at 60 °C.

### 2.4. Assembling MWCNTs-IL on the surface of silicon wafer

**Scheme 1:**
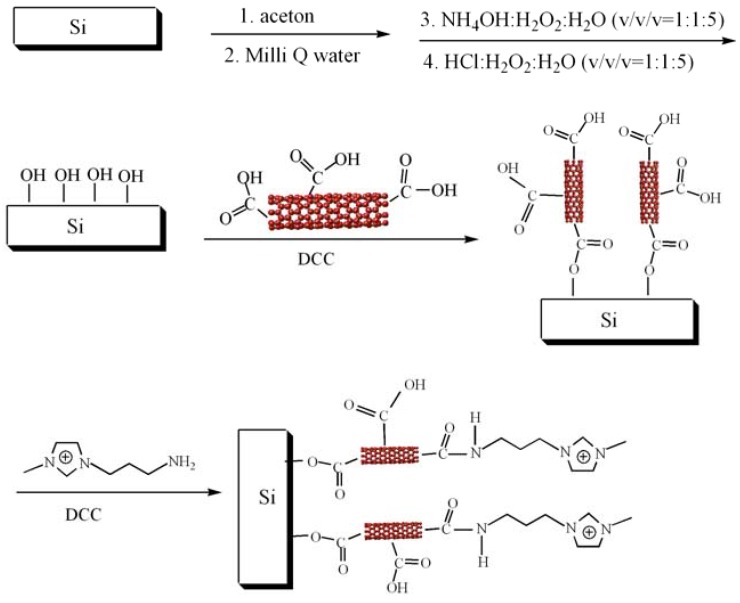
Procedure for assembling MWCNTs-IL on the surface of a silicon wafer.

The process used in this work is based on a previously reported method [26] and is shown in Scheme 1. Silicon wafers were cleaned in acetone and Milli Q water. Then they were chemically oxidized for 15 min at 80 °C in a mixture of NH_3_-H_2_O_2_-H_2_O (v/v/v=1:1:5). After the oxidation, silicon wafer was transferred into an 80 °C mixture of HCl-H_2_O_2_-H_2_O (v/v/v=1:1:5) for 15 min. During these two steps, hydroxide groups were produced on the surface of the silicon wafer (Si-OH). The silicon wafer was then washed with Milli Q water and dried with high purity nitrogen flow and put into the aqueous solution of activated MWCNTs for 24 h (denoted as Si-M). The silicon wafer modified with MWCNTs was cleaned by Milli Q water and then transferred to aqueous mixture of IL-NH_2_ and DCC for 24 h at room temperature, producing a Si substrate with IL-NH_2_ functionalized with MWCNTs arrays, Si-MIL.

### 2.5. Synthesis of MWCNTs-IL powder

Purified MWCNTs (MWCNTs-COOH, 0.0050 g), IL-NH_2_ (0.010 g), and DCC (0.010 g) in 10.0 mL of DMF were sonicated for 15 min and then held at 50 °C for 24 h under dynamic stirring, after which unreacted MWCNTs were removed by centrifugation. The black precipitate was dried under vacuum at 60 °C overnight.

### 2.6. Instruments and Measurements

Atomic force microscope (AFM, JEOL, JSPM-4200) tapping mode images of the samples were taken in air. Raman spectra were recorded using a Renishaw Ramascope 1000 B system equipped with a Leica DMLM microscope and connected to a charge-coupled device camera detector. The excitation wavelength was 780 nm (NIR diode laser) spectra were recorded in 180 degree from the Si-substrate at room temperature (23 ± 1 °C). Fourier transform infrared spectroscopy (FTIR) spectra were collected using a Bruker IFS 66/S Spectrometer equipped with an MCT-detector, KBr pellets were used for the MIL powder. The Si-MIL and Si-OH layers were studied by variable angle external reflection (Harrick Scientific, Seagull). A resolution of 4 cm^-1^ was used and 1,000 scans were recorded for each spectrum. The incident angle was set to 55°. Cyclic voltammetric scans were recorded using an Autolab electrochemical workstation (PGSTAT 100). A conventional three-electrode electrochemical cell was used with silicon wafer, a KCl-saturated silver-silver chloride (Ag|AgCl) electrode and a platinum wire as the working, reference and counter electrodes, respectively. All potentials reported here refer to the Ag|AgCl (sat. KCl) reference electrode.

## 3. Results and Discussion 

**Figure 1:**
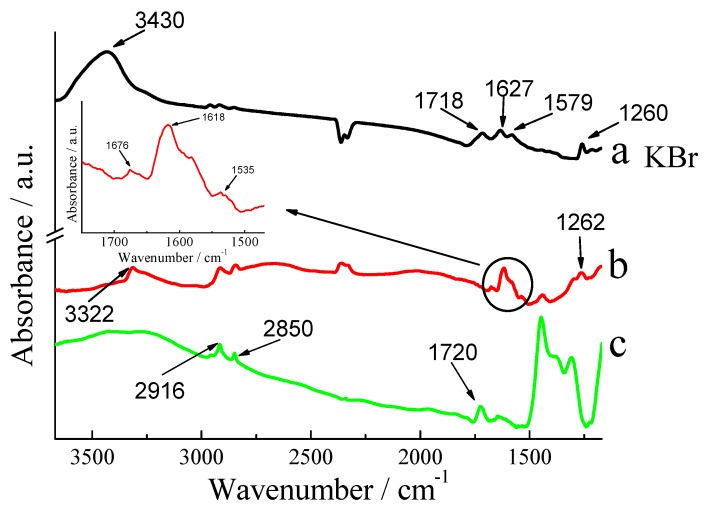
FTIR spectra of (a) MIL powder, (KBr), (b) Si-MIL (55°) and (c) Si-OH (55°).

FTIR reflection measurements were made in order to conform the formation of Si-MIL. The spectra are shown in Figure 1. Spectrum (a) is of MIL pressed into a KBr pellet, while spectrum (b) is the MIL assembled on a silicon wafer. Spectrum (c) is the silicon wafer modified with -OH groups. The absorption bands at 2,850 cm^-1^ and 2,916 cm^-1^ can be attributed to the CH_2_ and CH_3_ stretching vibrations from organic species on the Si surface originating from the chemical cleaning of the substrate [27]. The feature at 1,720 cm^-1^ can be assigned to oxygen in silicon [28]. The peak at 3,322 cm^-1^ in spectrum (b) is ascribed to the presence of O-H groups and is believed to result from either ambient atmospheric moisture tightly bound to the MWCNTs or as result of oxidation during purification of the raw material [29]. Peaks at 1,618 cm^-1^ and 1,535 cm^-1^ are the characteristic vibrations from the –C=O stretching and N-H vibration in amide I and amide II [30]. The peak at 1,676 cm^-1^ is assigned to the carboxylic C=O bond [31], which might come from the side surface of MWCNTs arrays [32]. When comparing the spectrum (b) with the spectrum (a), the peaks corresponding to the assembled film on the silicon wafer are shifted to lower wavenumbers, which can be interpreted as the effect of silicon wafer [30]. Based on these results, the formation of a bond between MWCNTs and IL can be confirmed.

**Figure 2:**
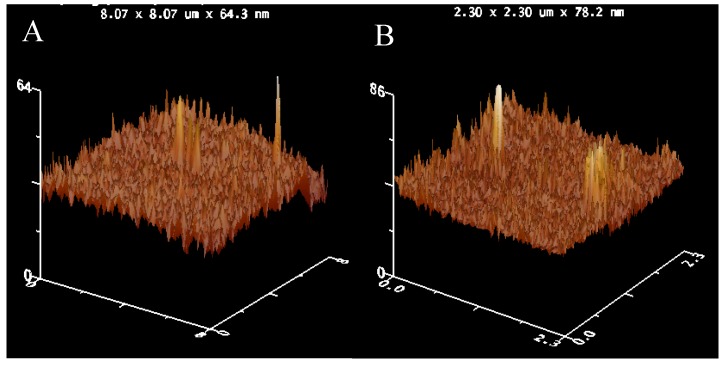
AFM images of (A) Si-M and (B) Si-MIL.

The morphologies of MWCNTs and MIL assembled on silicon wafer are shown in Figure 2. The AFM images show the MWCNTs alignments on the surface of silicon wafer (Figure 2A). The aligned tubes on the surface are not as individual tubes but as bundles. After modification with IL-NH_2_, the morphology of alignments was not affected (Figure 2B).

**Figure 3:**
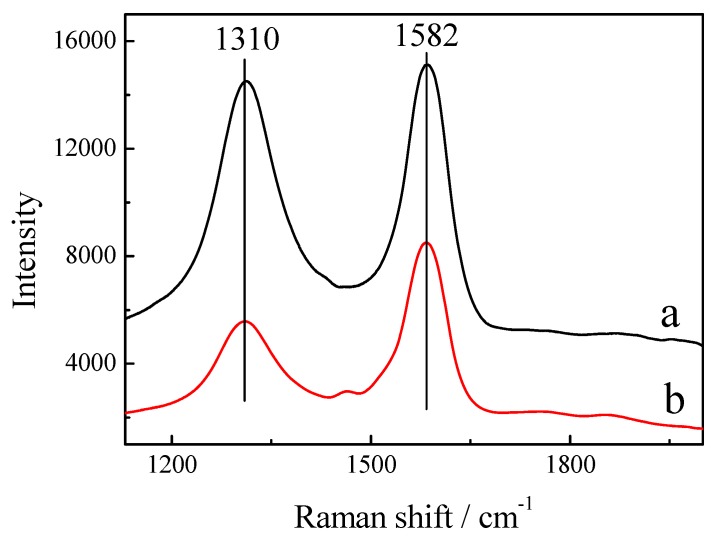
Raman spectra of (a) Si-M and (b) Si-MIL.

The assembled films of Si-M and Si-MIL were further characterized by Raman spectroscopy (Figure 3). The peaks at 1,310 cm^-1^ and 1,582 cm^-1^ can be attributed to the D-band and G-band from the CNTs, respectively [15]. The strong D-band corresponds to the defects in the curved graphene sheet, tube ends and staging disorder while the G-band is related to the graphitic hexagon-pinch mode. The R value (R = D/G) of Si-M (R = 0.96) changed remarkably after IL-NH_2_ modification (Si-MIL, R = 0.65). These results indicate that addition of IL on Si-M increased the disorder in the structure of MWCNTs and is another proof, in addition to the FTIR measurements, that the MWCNTs have successfully been modified by IL-NH_2_. On the other hand, the characteristic G band of the MWCNTs is still observed in the Raman spectrum even after functionalization with IL-NH_2_ indicating that the original properties of the MWCNTs are preserved in the final Si-MIL, as reported previously [6].

**Figure 4:**
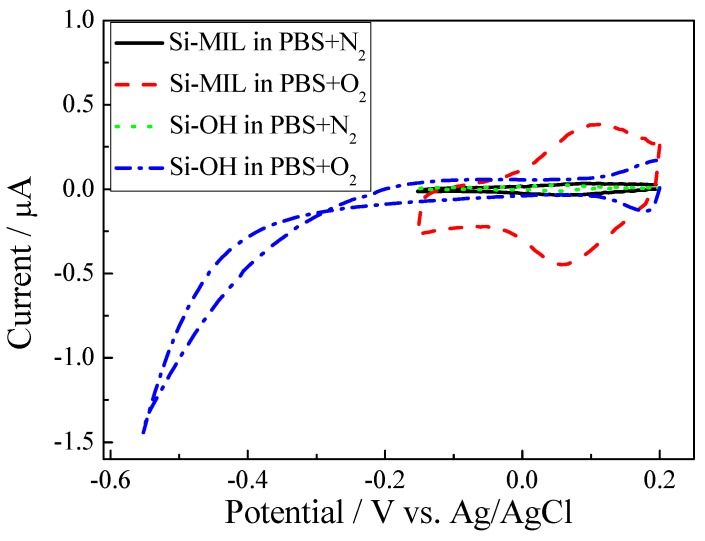
Cyclic voltammetric measurements of Si-MIL in PBS with O_2_ (dashed line), N_2_ (solid line), and Si-OH in PBS solution saturated with O_2_ (dotted line) and N_2_ (dashed dotted line), respectively. Scan rate: 0.05 V s^-1^.

The cyclic voltammograms (CV) at Si-MIL and Si-OH substrates in Phosphate Buffered Saline (PBS) solution saturated with oxygen and nitrogen are shown in Figure 4. The oxygen reduction peak at Si-MIL is observed at approximately 0.06 V. However, for the solution saturated with nitrogen, no reduction peak was detected at Si-MIL, which means that oxygen was electrocatalytically reduced at the substrate of Si-MIL. In order to confirm the electrocatalysis of MIL, a control experiment was made. For the substrate Si-OH, no reduction peak was detected in solutions either saturated with nitrogen or oxygen. Previous results from MIL powder drop-cast on glassy carbon electrode also showed electrocatalytic effect towards reduction of oxygen at -0.3 V [4]. In this work we have been able to demonstrate that the Si-MIL surface exhibits a clear catalytic activity to reduction of oxygen by shifting the reduction potential from -0.3 V to 0.06 V. Therefore, we can state that the assembled MIL layer studied in this work shows better electrocatalysis towards reduction of oxygen than the earlier studied drop-cast layer [4]. The improved catalytic activity of Si-MIL confirms that the tube ends of CNTs are the reactive sites [7,8].

## 4. Conclusions

MIL was assembled on the surface of silicon wafer by a simple chemical method. The sample was characterized by AFM, FTIR and Raman techniques and the formation of Si-MIL was confirmed. In addition, the CV measurement indicated that Si-MIL showed good electrocatalysis towards reduction of dissolved oxygen in aqueous solution. Moreover, it should be noticed that the electrocatalytic property of Si-MIL is better than that of MIL due to aligning the tubes leaving the high active of tube ends as the reaction sites.
